# 

**Published:** 1905-03-25

**Authors:** 


NOTICE TO CORRESPONDENTS.

All MSS., Letters, Books for Review, and other matters intended for the Editor should be addressed The Editor, "The Hospital," The Hospital Buildings, 28 & 29 Southampton Street, Strand, W.C.

The Editor cannot undertake to return rejected MS., even when accompanied by stamped directed envelope.

Notice to Advertisers and Subscribers.

All Advertisements, Orders for Copies of The Hospital, and Business Communications should be addressed to THE MANAGER (Not to the Editor), The Hospital, 28 & 29 Southampton Street, Strand, London, W.C.

The Cost of Subscription, post free, is as follows:—Per week, 3½d.; per quarter, 3s. 3d.; per half-year, 6s. 6d.; per annum, 13s. Foreign and Colonial:—Per annum, 19s. 6d., payable, in all cases, in advance.

**NOTICE.—Vol. XXXVI. of "The Hospital" (April to September 1904), in handsome cloth binding, is now on sale at the office of this Journal, price 10s. 6d. Cases for binding the Numbers contained in this Volume 2s. Index to Vol. XXXVI., 3½d., post free.**

**The Monthly part for March is now ready. Price 1s., by post, 1s. 3d.**

### MEDICAL APPOINTMENTS.

**W. H. Clayton-Greene**, M.B., B.C.Cantab., F.R.C.S.Eng., Surgeon in charge of Out-patients at St. Mary's Hospital. **F. Sherwill Dawe**, M.D., B.Sc.Lond., Casualty Physician to St. Mary's Hospital, London. **J. P. Garlick**, M.B., Ch.B.Vict., Assistant House and Visiting Surgeon at the Stockport Infirmary. **Cecil H. Leaf**, F.R.C.S.Eng., Honorary Surgeon to the Cancer Hospital, London. **George Barton McCaul**, M.B., B.Ch., B.A.O.Dub., L.M.Rotunda, Resident Obstetric Officer at the Bristol Royal Infirmary. **G. H. Makins**, C.B., F.R.C.S.Eng., to the Consulting Staff of the Bolingbroke Hospital, Wandsworth Common, S.W. **W. E. Miles**, F.R.C.S.Eng., Honorary Surgeon to the Cancer Hospital, London. **C. H. Mollinson**, M.B., Lecturer of Forensic Medicine at the Melbourne University, Australia. **R. T. Newstead**, Lecturer in Economic Entomology and Parasitology in the Liverpool School of Tropical Medicine. **W. Laidlaw Purves**, M.D., M.R.C.P., Consulting Ophthalmic Surgeon, Hospital for Epilepsy and Paralysis and other Diseases of the Nervous System, Maida Vale, London, W. **A. Lewin Sheppard**, M.B., B.S.Durh., Casualty Officer to the Bristol Royal Infirmary. **Robert Shingleton Smith**, M.D.Lond., F.R.C.P.Lond., Honorary Consulting Physician to the Bristol Royal Infirmary. **StClair Thomson**, M.D., F.R.C.P., F.R.C.S., Physician for Diseases of the Throat in King's College Hospital. **Leonard Williams**, M.D.Glasg., M.R.C.P.Lond., Honorary Physician to the French Hospital and Dispensary, London. **Patrick Watson Williams**, M.D.Lond., M.R.C.S., Honorary Physician to the Bristol Royal Infirmary.

**"The Hospital" Institutional Library.**

**"Hospitals and Asylums of the World." 4 Vols., with a Portfolio of Plans, complete £12 12s.**

**"Burdett's Hospitals and Charities, 1904." 5s. net.**

**"Burdett's Official Nursing Directory." 8s. net.**

**"Cottage Hospitals, General, Fever, and Convalescent." 10s. 6d.**

**"Uniform System of Accounts for Hospitals and Public Institutions." New Edition. 4s. net.**

**"Hospital Expenditure: The Commissariat." 2s. 6d.**

**"A Legal Handbook for the Use of Hospital Authorities." 2s. 6d.**

**"The Cottage Hospital Case Book or Register of Patients." 10s. and 12s. 6d.**

**All these are published by the Scientific Press, Ltd., and may be obtained through any bookseller, or direct from the publishers, 28 and 29 Southampton Street, Strand, London, W.C.**

### The Modern Treatment of Consumption.

**TENDENCIES TO CONSUMPTION: HOW TO COUNTERACT THEM.** By J. D. MORTIMER, M.R.C.S. 2/6 post free.

**THE MODERN NURSING OF CONSUMPTION.** By Dr. Jane Walker, Medical Superintendeut East Anglian Sanatorium. 1/- net, 1/1 post free.

Works by **T. N. KELYNACK, M.D.,** Physician in Charge, Northwood Hospital for Consumption.

**SANATORIUM TREATMENT OF CONSUMPTION.** 6d. net, 7d. post free.

**SELECTION OF CONSUMPTIVE CASES FOR SANATORIUM TREATMENT.** 6d. net, 7d. post free.

**A SPECIAL CHART FOR CONSUMPTIVE CASES.** Especially designed to meet the latest requirements of Modern Consumption Treatment. The size of the Chart is 15¼ in. by 11¾ in., printed on good paper, or it may be obtained on buckram for cases that must be kept constantly in the open air. Sample copy, 2½d. post free. Special quotations for quantities.

**CHART HOLDER.** Specially constructed for the above Chart. Japanned tin, white or black. Price 1/6 each, 1/9 post free. Price for quantities on application.

**CASE PAPERS FOR RECORDING THE HISTORY AND PROGRESS OF CASES.** Consisting of 4 pp., foolscap size. With Diagrams for defining the stages of the disease. Sample copies, each 2½d. post free. Special quotations for quantities. Continuation Papers, consisting of an additional 4 pp., also supplied. Prices on application.

**PATHOLOGICAL REPORTS.** Specially drawn up for reporting cases in the most concise and practical manner. Consisting of 4 pp., foolscap size. Sample copies, each 2½d. post free. Special quotations for quantities. This Chart and Case Papers enable Sanatoria to record their cases on a uniform basis, thus rendering the information so preserved of immense value in scientific research.

*TUBERCULOSIS*, the Journal of the National Association for the Prevention of Consumption, says: "A series of Charts and Report Forms admirably adapted for recording cases in Sanatoria for Consumptives."

**POCKET SEAT FOR CONSUMPTIVES.** The size of a large pocket-book when folded. Weight about 4 oz. This will be found an indispensable companion to the consumptive patient as a protection against chills through sitting on damp seats, &c. Price 1/3 post free.

***All Requisites for the use of Modern Consumption Treatment and Nursing supplied.***

THE SCIENTIFIC PRESS, LTD.
28 & 29 Southampton Street, Strand, London, W.C.

### Text-Books for Nurses.

| | | Post free |
|---|---|---|
| Watson. A Handbook for Nurses. | 5/- net | 5/4 |
| Colton. The Human Body: Practical Physiology and Hygiene ... ... | | 5/- |
| Miles. Surgical Ward Work | 3/6 net | 3/10 |
| Creighton Hale. Art of Massage ... ... | | 6/- |
| Hampton. Nursing ... ... | 7/6 net | 7/10 |
| Stephenson. Ophthalmic Nursing. | 3/6 net | 3/10 |
| Macleod Yearsley. Nursing in Diseases of Throat, Nose, and Ear ... ... | | 2/6 |
| Watson. A Complete Handbook of Midwifery ... ... ... ... | 6/- net | 6/4 |

| | | Post free |
|---|---|---|
| McAdam Eccles. Elementary Anatomy and Surgery ... ... ... ... ... | | 2/6 |
| Marshall. Elementary Physiology ... | | 2/- |
| Johnson Smith. Practical Guide to Surgical Bandaging and Dressings ... | | 2/- |
| First-Aid to the Injured and Management of the Sick ... ... ... ... | | 3/6 |
| Morten. Nurses' Pronouncing Dictionary ... ... ... ... | | 2/- |
| Sequeira. Elementary Treatise on the Light Treatment ... ... | 2/6 net | 2/9 |
| Lückes. Hospital Sisters and their Duties ... ... ... ... ... | | 2/6 |

***Send for Complete Catalogue of Nursing Manuals and Samples of Temperature Charts.***

THE SCIENTIFIC PRESS, LTD.
28 & 29 Southampton Street, Strand, London, W.C.

### WORKS FOR NURSES.

**LESSONS ON MASSAGE.** By Mrs. MARGARET D. PALMER, Manager of the Massage Department and Instructor of Massage to the Nursing Staff of the London Hospital. Second Edition, enlarged and revised. With 118 Illustrations. Price 7/6 net.

**A MANUAL FOR STUDENTS OF MASSAGE.** By Miss M. A. ELLISON, L.O.S. Second Edition, edited by Miss G. MANLEY, pp. xii + 126. With 50 original Illustrations. Price 3/6 net.

**NOTES ON HOME NURSING.** By Miss D. M. GOLDIE, Lecturer on Nursing, Hygiene, &c., to the County Councils of London, Surrey, &c. Price 1/6 net.

**MIDWIFERY FOR MIDWIVES.** Designed to cover the requirements of the Examining Board authorised by the Midwives Act. With Appendix on Examinations. By W. DENISON WIGGINS, M.R.C.S., L.R.C.P., &c. Price 3/6 net.

**MOVABLE ATLAS OF THE ANATOMY AND PHYSIOLOGY OF THE CHILD.** With Explanatory Text. By D'ARCY POWER, M.A.Oxon, M.B., F.R.C.S. Price 3/- net.

**AIDS TO GYNÆCOLOGY.** By A. S. GUBB, M.D., M.R.C.S. 10th Thousand. Pp. 153, with 29 Figs. Price 2/6.

**AIDS TO PHYSIOLOGY.** By PEYTON T. B. Beale, F.R.C.S., Eng., Examiner in Physiology to the Society of Apothecaries; Lecturer in Physiology and Histology, Women's Department, King's College. New Edition. Pp. 240. With 48 Illustrations. Price 3/6.

**A MANUAL FOR HOME NURSING.** By BERNARD MYERS, M.D., M.R.C.S., Surgeon and Lecturer to St. John's Ambulance Association. Price 2/6 net.

**THE FEEDING AND HYGIENE OF INFANTS AND CHILDREN.** By JOHN McCAW, M.D., M.R.C.P., Physician to the Belfast Hospital for Children. Price 2/6.

**QUESTIONS AND ANSWERS ON MIDWIFERY FOR MIDWIVES.** By A. B. CALDER, M.B., C.M.Ed., M.R.C.S.Eng., Lecturer on Obstetrics, Fulham School of Midwives. Price 1/6 net.

**AFTER-TREATMENT OF OPERATIONS.** By P. LOCKHART MUMMERY, B.A., M.B., F.R.C.S., Demonstrator of Operative Surgery, St. George's Hospital. Second Edition. Pp. viii + 221, with 29 Figs. Price 5/- net.

**AIDS TO OBSTETRICS.** By S. NALL, M.B., M.R.C.P. 23rd Thousand. Pp. 146. Price 2/6.

*A Catalogue of Books for Nurses post free on application.*

London: BAILLIÈRE, TINDALL & COX, 8 Henrietta Street, Covent Garden.

